# Mothers’ Adverse Childhood Experiences Moderate the Association between Maternal Anger and Children’s Externalizing Symptoms

**DOI:** 10.3390/children11081002

**Published:** 2024-08-16

**Authors:** Tiago Castro, Rita Pasion, Carla Antunes, Francisca Alves, Inês Jongenelen, Diogo Lamela

**Affiliations:** HEI-Lab—Digital Human-Environment Interaction Labs, Lusófona University, 4000-098 Lisbon, Portugal

**Keywords:** adverse childhood experiences, anger, externalizing symptoms, child maltreatment, adversity

## Abstract

Background/objectives: This study examined the association between maternal anger, children’s externalizing symptoms, and the moderating impact of maternal adverse childhood experiences (ACEs) in the context of intimate partner violence (IPV). The primary objective was to investigate whether maternal ACEs alter the link between maternal anger and children’s externalizing symptoms, guided by theoretical frameworks such as the stress sensitization and stress steeling models. Methods: A sample of 159 Portuguese mothers exposed to IPV participated in the study, completing a protocol of self-report measures. Measured variables included maternal anger, ACEs, children’s externalizing symptoms, and IPV. Results: Results indicate a significant moderation effect of ACEs on the association between maternal anger and externalizing symptoms at low levels of ACEs. Conversely, at moderate and high ACEs levels, no statistically significant association exists between maternal anger and children’s externalizing symptoms. Conclusions: Clinical implications emphasize the importance of tailored parenting interventions to prevent externalizing symptoms in children, integrating diverse emotion regulation strategies while considering the impact of maternal ACEs.

## 1. Introduction

Anger is a complex emotional state arising from perceived threats or wrongdoing and emerges when individuals encounter obstacles to goal achievement, resulting in negative feelings linked to perceived transgressions [1,2]. Manifesting in various forms, such as emotion, mood, or affective temperament, anger varies in intensity, duration, and frequency and is intricately tied to aggression and violence [3]. Physiologically, anger encompasses arousal states ranging from mild irritation or annoyance to intense fury and rage [4]. In social situations perceived as threatening or unpleasant, anger acts as an emotional response, often triggered by perceived obstacles or unfair decisions [5]. 

The impact of anger on interpersonal dynamics, particularly within family contexts, is underscored by substantial research that establishes an association between maternal anger and adverse outcomes in children [6]. Specifically, this association manifests in the increased likelihood for children to display externalizing symptoms, a consequence attributed to challenges children face in regulating negative emotions [7,8,9]. The socialization of emotion model highlights the role of parents as socioemotional models for their children, serving as a key reference for understanding emotions in a social context [10,11]. As such, parents’ emotional expressions, including anger, play a pivotal role in shaping children’s socioemotional functioning by providing templates for their own understanding and regulation of emotions [10,12]. More specifically, as parents serve as models for diverse emotional responses, their expressions of emotions modulate how children interpret and respond to emotional stimuli in their social environment [13]. Consequently, children not only acquire the skills to express emotions but also develop insights into situations where specific emotions are considered acceptable or appropriate [13,14]. Therefore, mothers exhibiting dysregulated anger may inadvertently model maladaptive coping mechanisms for dealing with perceived threats [15,16]. Offspring of mothers with elevated anger expression levels may exhibit a low threshold for experiencing anger, demonstrating anger/temper outbursts and heightened irritability [17,18]. Given that mothers may be engaged in regulating their own anger, they are likely to be less available to address their children’s negative affect and assist them in developing coping strategies [19]. In the face of continual maternal expressions of anger, children are also more susceptible to developing heightened reactivity to negative emotional stimuli, including anger, sadness, and fear [20,21]. For instance, these children are more likely to display hostile attribution bias when processing social stimuli, interpreting ambiguous and neutral social cues as hostile or threatening [22]. This distortion in the child’s understanding of others’ intentions results in negative interpretations of others’ behaviors, social difficulties, and rejection by peers [23]. These challenges, in turn, may contribute to the expression of aggressive behaviors towards both peers and figures of authority [24]. Taken together, the interplay between parents’ modeling of anger expression, children’s difficulties in regulating anger expression, and coercive (i.e., negative, hostile, or aggressive) mother–child interactions may culminate in the emergence of externalizing symptoms [25,26,27].

The association between maternal anger and children’s externalizing symptoms may be amplified in environments characterized by high stress, such as those involving exposure to IPV. IPV is strongly associated with higher risk of mental health problems in mothers, including difficulties in emotion regulation, particularly in managing anger [28]. Research indicates that mothers who have experienced IPV are more likely to struggle with regulating their emotions during interactions with their children, compared to mothers with no history of IPV [29]. In parallel, children exposed to IPV—whether directly or indirectly—are at a heightened risk for developing emotional and behavioral problems, such as increased difficulties with emotion regulation and more pronounced externalizing symptoms [30]. Consequently, examining maternal anger within the context of IPV may uncover variations in children’s externalizing behaviors that might not be identifiable in community-based populations.

### The Moderating Role of Maternal Adverse Childhood Experiences

Given the growing body of literature demonstrating the direct consequences of maternal anger on children’s externalizing symptoms [31,32,33], the next step entails the identification of factors that might alter this relationship. This investigation is crucial for gaining insights into an association that may be more complex than linear and potentially mitigating the risks associated with maternal anger on the developmental outcomes of their children. Existing research has predominantly focused on the buffering effects of proximal individual factors, such as children’s sex and maternal cortisol levels [34]. However, the potential moderating role of distal social factors, including mothers’ ACEs, remains an unstudied venue.

This study specifically directs attention to mothers’ adverse childhood experiences (ACEs) as a potential variable that may contribute to explaining interindividual variability in the association between maternal anger and children’s externalizing symptoms. A growing body of research, grounded in the cumulative risk perspective, suggests that childhood adversity may heighten the likelihood of subsequent adverse experiences in adulthood, such as intimate partner violence [35], and is temporally associated with transdiagnostic mechanisms of adult psychopathology, including anger, hostility, and rumination [36]. 

Despite the increasing awareness of these associations, there is a notable scarcity of information regarding how ACEs may modify the association between maternal anger and children’s externalizing symptoms. The exploration of the moderating role of ACEs is framed within two distinct theoretical perspectives—the stress sensitization model and the stress steeling model. 

The stress sensitization model posits that early adversity may contribute to enduring emotional distress and psychopathology by heightening an individual’s overall sensitivity to stress across the lifespan [37,38]. Initially, major stressors play a significant role in triggering affective episodes, but over time, even minor stressors can elicit similar responses. This progression highlights that both the severity and frequency of early adversity are crucial in shaping long-term stress sensitivity and vulnerability to psychopathology [38]. As such, under this model, mothers’ ACEs may exacerbate the effect of maternal anger expression on externalizing symptoms in their children. In contrast, the stress steeling model proposes that moderate stress levels can facilitate the development of coping mechanisms and resilience, “steeling” individuals against future stress [39,40,41]. This hypothesis implies that early exposure to mild stressors may afford protective advantages to children by potentially diminishing subsequent distress and vulnerability, consequently enhancing their capacity for emotion regulation in stressful situations [41,42]. In this context, mild ACEs may mitigate the deleterious effects of maternal hostile behaviors on children’s externalizing behavior by enhancing maternal emotional regulatory competencies, thereby tempering hostile behaviors and associated negative affective states. Research has provided some evidence to support both stress sensitization [43,44,45] and stress steeling models [46,47].

To date, research has primarily focused on examining how the sensitization and steeling effects of accumulated past life events mitigate the impact of recent stressor exposure on later mental health outcomes. Notably, there is a lack of research investigating whether ACEs may act as moderators in the association between maternal emotional states and children’s socioemotional functioning. The current study aims to address this gap in the literature by testing maternal ACEs as a moderator of the association between mothers’ anger and children’s externalizing symptoms, examining whether maternal ACEs function in accordance with the hypothesized stress sensitization model or stress steeling model.

## 2. Method

### 2.1. Participants

The study involved 159 mothers residing in Portugal, each with children aged between 6 and 10 years old. All these mothers had experienced police-reported intimate partner violence within the past year. Recruitment took place through contact with child protection services and shelters designed for victims of domestic violence across the entire country. Criteria for participation included: (a) the mother being at least 18 years old, (b) the child residing with the mother either at home with the abusive partner or in a shelter home, and (c) the child not having a diagnosed cognitive or sensory disorder.

The average age of the mothers was 36.3 years (*SD* = 7.5; range = 21–58 years). Among the participants, 56.6% were married or cohabiting, 23.2% were divorced or separated, and 20.2% were either single or widowed. The majority of the mothers identified as Caucasian (95%), with 4.4% being Black, and 0.6% belonging to other ethnicities. Regarding education, 53.5% of the mothers had less than nine years of schooling, 36.4% completed the 9th year of schooling, 8.2% finished high school, and only 1.9% held a higher education degree. A significant portion of the mothers (75%) were unemployed, and 48% received public social assistance. The focal child’s average age was 7.3 years (*SD* = 1.94), with 54% being boys (*n* = 87).

### 2.2. Measures

#### 2.2.1. Mother’s Anger

Mothers’ anger was assessed using the hostility scale of the brief symptom inventory [48] (BSI). This 5-item scale measures individuals’ feelings of annoyance and irritability, urges to break things, frequent arguments, and uncontrollable outbursts of temper. Respondents were asked to answer regarding their levels of distress over the previous two weeks, using a 5-point Likert scale (from 0 ‘not at all’ to 4 ‘extremely’). Higher scores reflected higher anger-related problems. Previous empirical work has shown that the BSI hostility scale is a reliable and valid measure for screening anger problems [49,50]. The Portuguese version of the depression scale of the BSI revealed good internal consistency [51]. In the current sample, Cronbach’s alpha was 0.82.

#### 2.2.2. Mother’s Adverse Childhood Experiences

The Adverse Childhood Experiences (ACEs) Questionnaire [52] is a retrospective self-report instrument designed to assess exposure to various adverse experiences during childhood. The questionnaire comprises 10 items, each pertaining to distinct categories of adversities, including physical abuse, emotional abuse, sexual abuse, physical neglect, emotional neglect, household substance abuse, household mental illness, parental separation or divorce, witnessing domestic violence, and having an incarcerated household member. The items were dichotomized using a binary response format, with a score of 1″ indicating the presence of an adverse experience and a score of 0″ indicating its absence. The cumulative score represents the overall burden of adverse experiences, with a higher score indicating a greater extent of childhood adversity. The Portuguese version of the depression scale of the BSI revealed good psychometric properties. In the current sample, the Cronbach’s alpha was 0.70.

#### 2.2.3. Child Externalizing Symptoms

Externalizing problems were assessed using the hyperactivity/inattention and conduct problems subscales of the Strengths and Difficulties Questionnaire—Parent Form (SDQ) [53]. Each of these 5-item subscales was completed by mothers using a three-point Likert-type scale, ranging from 0″ (not true) to 2″ (very true), reflecting the degree to which each symptom characterized their child over the last six months. The composite externalizing symptoms score was derived by summing the scores of these two subscales, with higher scores indicating greater externalizing symptoms. The Portuguese version of the SDQ demonstrated satisfactory psychometric properties [54]. In the current study, Cronbach’s alpha was 0.72.

#### 2.2.4. Intimate Partner Violence

Intimate partner violence was assessed using the 39-item Conflict Tactics Scale-2 [55]. Participants reported their exposure to physical (including injuries), psychological, and sexual violence perpetrated by an intimate partner in the last 12 months. Responses were made on a 6-point Likert-type scale ranging from ‘never’ to ‘more than 20 times’. Higher scores reflected higher intimate violence victimization. The Portuguese version of the depression scale of the BSI revealed good internal consistency [56]. Internal consistency for the CTS2 total score in the current study was 0.87.

### 2.3. Procedures

Participants were recruited through collaboration with 12 child protective services and 37 shelters. Potential participants who met the inclusion criteria were approached by professionals from these institutions, who provided detailed information regarding the study’s objectives and procedures. Following the acquisition of written informed consent, female members of the research team administered the comprehensive assessment protocol to both mothers and children within the confines of child protection services or shelter facilities. The informed consent process not only conveyed the study’s objectives and procedures but also assured participants of data confidentiality and safety measures. It was explicitly communicated that professionals associated with child protective services or shelters would not be involved in data collection, analysis, or storage. Additionally, participants were assured that their responses would not impact the nature of their relationship or support from these organizations. To uphold ethical standards, participants were explicitly informed of their right to non-participation or withdrawal from the study without any adverse consequences. The assessment protocols were administered individually. Mothers, as a token of appreciation for their participation, received vouchers from a local department store. The research project received ethical approval from the Portuguese National Data Protection Commission (10290/2014).

### 2.4. Analytic Strategy

Firstly, as part of our rigorous data management process, we thoroughly reviewed all coding and reverse-coding procedures to ensure their accuracy. Secondly, preliminary bivariate correlations were conducted to determine the associations between the variables. Moderation analyses were then carried out using the PROCESS macro in SPSS-v28, employing an ordinary least squares approach and a bias-corrected bootstrap method with 5000 samples to estimate conditional effects. In the moderation model to predict children’s externalizing symptoms, maternal anger was entered as the independent variable, ACEs as the moderator variable, and IPV as the covariate. To address multicollinearity, mean-centering of all continuous variables was performed. Interaction terms were created by computing the product of the two mean-centered main effects and were subsequently entered into linear regression analyses. To probe statistically significant interaction effects, we first examined the Δ*R*^2^ value and then calculated the conditional effects at different levels of the moderator (16th, 50th, and 84th percentiles).

## 3. Results

Table 1 presents the means and standard deviations of the study’s main variables. Correlation analyses revealed that the study’s variables presented significant associations in the expected directions, with the exception of externalizing symptoms, which showed no significant correlations with most of the remaining variables.

**Figure 1:**
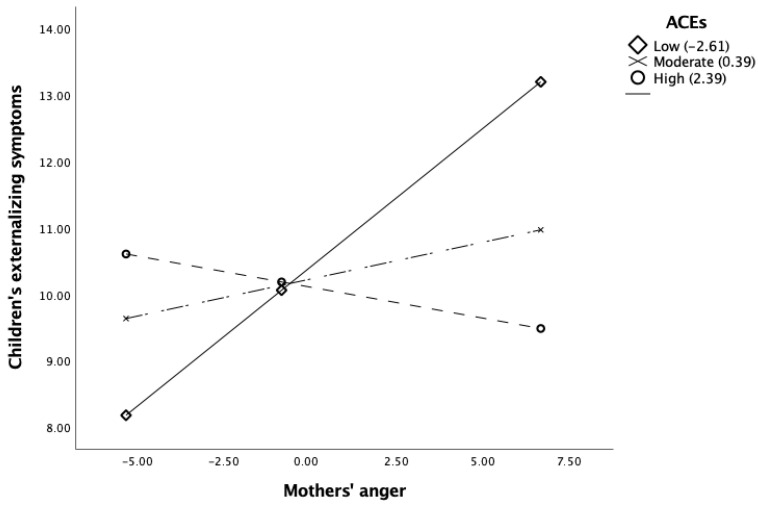
Conditional effects of maternal ACEs on the association between mothers’ anger and children’s externalizing symptoms.

The overall moderation model was statistically significant, *R*^2^ = 0.08, *F*(4, 154) = 3.34, *p* < 0.001 (Table 2). Firstly, we found a direct significant relationship between mothers’ anger and children’s externalizing symptoms, *b* = 0.15, *p* < 0.05. Secondly, the moderation effect of mothers’ ACEs on the association between mothers’ anger and externalizing symptoms was statistically significant, Δ*R*^2^ = 0.06, Δ*F*(1, 154) = 10.70, *p* < 0.001. The interaction term for mothers’ anger and ACEs explained a substantial portion of the variance in externalizing symptoms, *b* = −0.10, *p* < 0.001. The conditional effect analysis showed that at low (16th percentile) levels of ACEs, the association between mothers’ anger and externalizing symptoms was positive and statistically significant (*b* = 0.42, *p* < 0.001) (Figure 1). At moderate and high levels of ACEs, there was no statistically significant association between mothers’ anger and children’s externalizing symptoms.

## 4. Discussion

This study aimed to investigate the association between maternal anger and child externalizing symptoms, including mothers’ ACEs as a moderator, in a sample of Portuguese mothers exposed to intimate partner violence (IPV) and their children. Our first findings showed that maternal anger had a positive association with children’s externalizing problems. This aligns with prior studies conducted with preschool, school-aged, and adolescent children, suggesting that a temporal association between maternal emotional expression of anger is linked to various externalizing-related disorders, both in mothers from the general community and those with social vulnerability or clinical problems [57,58,59,60].

Nonetheless, mothers’ ACEs moderated this relationship, particularly when considering the decomposition of conditional effects. In the conditional effect analysis of the data, the interaction between maternal anger and children’s externalizing symptoms was more pronounced when mothers had lower ACEs. However, at moderate and high levels of ACEs, the impact of maternal anger on children’s externalizing symptoms was not statistically significant, indicating a weakened or absent association between maternal anger and children’s symptoms in mothers with moderate and high levels of ACEs. 

The findings, which indicate that children of mothers with lower ACEs are more responsive or sensitive to the impact of maternal anger, and the absence of an association between maternal anger and children’s externalizing symptoms at high levels of mothers’ ACEs, are surprising and counterintuitive, especially in light of the stress sensitization model [38]. According to this model, one would anticipate a gradual increase in the impact of maternal anger on externalizing symptoms as mothers’ ACEs increase, given the expected linear association between early adversity, psychopathological outcomes, and an individual’s overall sensitivity to stress across the lifespan [37,38]. Indeed, we found that in the subgroup of mothers with low ACEs, higher levels of maternal anger were associated with a more pronounced increase in children’s externalizing symptoms. However, this linear association did not generalize to other levels of the moderator (i.e., 50th and 84th percentiles), suggesting that theoretical assumptions from the stress steeling model may better fit our results. 

According to the stress steeling model [39,41], a curvilinear, U-shaped effect better represents the complex associations between the variables under study. The main assumption is that moderate stress levels can facilitate the development of coping mechanisms that will protect individuals against future stressful events [41,42]. As such, this model accommodates the idea that mothers with moderate ACEs can exhibit a reduction in anger and stress reactivity compared to mothers with low ACEs, leading to a non-association with children’s externalizing symptoms. Therefore, the lack of association in our study between maternal anger and children’s externalizing symptoms at moderate levels of mothers’ ACEs in our study seems to be in line with the stress steeling model. Mothers with moderate levels of ACEs, exposed to moderate levels of stress during childhood, may have developed effective coping strategies and mechanisms that enable the regulation and adaptive expression of anger. This may contribute to children’s comprehension and regulation of their emotions, influencing the modulation of anger expression and subsequently decreasing the likelihood of externalizing symptoms. When considering the extreme endpoints of the U-shaped curve (16th and 84th percentiles), it can be hypothesized that both low and high levels of ACEs would produce a comparable outcome: an increased impact of maternal anger on children’s externalizing symptoms. This expectation arises because when distressing events deviate from the mild-optimal point, individuals may struggle to develop effective strategies for coping with emotional regulation difficulties in stressful situations. These difficulties may stem from a lack of concrete social models to guide responses to such situations or from exposure to an extreme environment where the interplay between parental modeling of anger expression and unavailability to address children’s needs regarding emotional expression compromises the acquisition of socioemotional skills by children [25,26,27]. Although the effect was significant at low levels, the absence of an association between maternal anger and children’s externalizing symptoms at high levels of mothers’ ACEs requires further investigation in future studies. Overall, these results are tentative and should first be replicated to understand their empirical consistency, as they might reflect a possible delayed-onset effect on children’s externalizing symptoms or a desensitization effect not yet systematically described in the literature.

This study has limitations that require consideration when interpreting the results. Firstly, we relied on self-reported data, which heavily depends on the accuracy of participants’ recall memory and, therefore, does not eliminate the risk of reporting bias. Additionally, children’s externalizing symptoms were exclusively reported by their mothers. The inclusion of observational assessment methods and interviews could have bolstered the reliability of the data. The incorporation of multimethod approaches might have improved measurement accuracy and alleviated potential shared method variance. Moreover, the study was exclusively conducted with mothers who experienced intimate partner violence, limiting the generalizability of the results, as women exposed to IPV present significantly higher exposure to ACEs than women from community settings. Another limitation of this study was that we included only IPV as a covariate in our analysis. While IPV is a significant factor, other variables such as additional current adversities, maternal mental health, and parenting style, which may also influence children’s externalizing behavior, were not considered. Future research should incorporate these additional covariates to provide a more nuanced understanding of the factors contributing to children’s behavioral outcomes. Finally, the study did not account for potential temporal variations in the moderating effect of ACEs on the association between maternal anger and children’s externalizing symptoms. Unfortunately, the cross-sectional design of the present study precludes exploring the differential impact of these changes over time on our findings.

Future studies should consider these limitations and explore alternative potential moderators and additional mediators. This could involve investigating specific emotion-regulation skills in response to negative-emotion-eliciting situations (e.g., rumination and suppression), examining constructive and destructive marital conflict resolution strategies, and evaluating maternal coping skills [61,62]. Additionally, exploring the role of coparenting dynamics as a moderator could provide valuable insights, as the quality of coparenting relationships may influence how parents’ emotional regulation impacts children’s behavioral outcomes [63]. Methodologically, future studies should incorporate a bidirectional analysis of maternal anger and children’s externalizing symptoms. This approach would facilitate an exploration of how ACEs might significantly moderate coercive and bidirectional mother–child interactions.

### Clinical Implications

Our findings contribute to an enhanced understanding of the influence of ACEs on the relationship between maternal anger and children’s externalizing symptoms. This contribution can further enrich the ongoing discourse regarding the challenges inherent in defining various forms of child maltreatment and their repercussions on children’s development and well-being [64]. Moreover, this study underscores the pivotal role of mothers’ past experiences in shaping their current levels of anger, elucidating the variability in their children’s externalizing symptoms. The integration of diverse emotion regulation strategies into parenting intervention programs could assist mothers in mitigating the impact of ACEs on their anger, potentially preventing their children’s exposure to maternal anger and, consequently, forestalling the development of externalizing symptoms in children. Our results also highlight the importance of incorporating resilience-building strategies into parenting programs for IPV-affected families, while also emphasizing the need for targeted, trauma-informed approaches to address the interplay between maternal anger and ACEs. By fostering emotional resilience in children, such interventions could buffer against the negative impacts of maternal anger, reducing the risk of externalizing behaviors and promoting healthier developmental outcomes in these vulnerable populations. Adopting a trauma-informed approach can further enhance the effectiveness of these interventions, ultimately supporting more adaptive mother–child interactions and improving outcomes for children exposed to IPV.

## Figures and Tables

**Table 1:** Descriptive statistics and bivariate correlations among main variables.

	M (SD)	1.	2.	3.
1. Externalizing symptoms	9.61 (4.13)	–		
2. Mothers’ anger	7.32 (5.02)	0.14	–	
3. Mothers’ ACEs	4.61 (2.41)	0.06	0.35 ***	–
4. Intimate partner violence (IPV)	23.39 (5.29)	0.10	0.30 ***	0.25 **

** *p* < 0.01; *** *p* < 0.001.

**Table 2:** Moderating effects of mothers’ ACEs on the association between mothers’ anger and children’s externalizing symptoms.

	*b*	*se*	*t*	LLCI	ULCI
Mothers’ anger	0.15	0.07	2.07 *	0.0071	0.2958
Mothers’ ACEs	−0.06	0.15	−0.39	−0.3545	0.2358
Mothers’ anger × Mothers’ ACEs	−0.10	0.03	−3.27 ***	−0.1642	−0.0406
Intimate partner violence (IPV)	−0.03	0.08	−0.39	−1.99	0.1323
**Conditional effects** **Mothers’ ACE’s**	***b***	***se***	***t***	**LLCI**	**ULCI**
Low (−2.61)	0.42	0.12	3.53 ***	0.1841	6527
Moderate (0.39)	0.11	0.07	1.54	−0.0309	0.2534
High (2.39)	−0.09	0.09	−0.98	−0.2813	0.0944

***Note.*** LLCI = lower limit confidence interval; ULCI = upper limit confidence interval. Low (−2.61) represents two standard deviations below the mean of the moderator variable. Moderate (0.39) represents the mean of the moderator variable. High (2.39) represents two standard deviations above the mean of the moderator variable. * *p* < 0.05; *** *p* < 0.001.

## Data Availability

Dataset available on request from the authors.
